# The Use of Tobacco Industry Vaping Products in the UK and Product Characteristics: A Cross-Sectional Survey

**DOI:** 10.1093/ntr/ntab253

**Published:** 2021-12-09

**Authors:** Elliot J Cornish, Leonie S Brose, Ann McNeill

**Affiliations:** Addictions Department, Institute of Psychiatry, Psychology and Neuroscience, King’s College London, London, UK; Addictions Department, Institute of Psychiatry, Psychology and Neuroscience, King’s College London, London, UK; SPECTRUM Consortium, London, UK; Addictions Department, Institute of Psychiatry, Psychology and Neuroscience, King’s College London, London, UK; SPECTRUM Consortium, London, UK

## Abstract

**Background:**

Tobacco industry (TI) companies have entered the UK e-cigarette (“vaping”) market in recent years. However, their motives and ambitions are unclear. This study explored how popular TI vaping products are and who uses them, and how they differ from independent products.

**Methods:**

Secondary analysis of data from a longitudinal web-based survey of smokers, ex-smokers, and vapers (*n* = 3883) in the UK in 2019. The main study sample consisted of daily and nondaily vapers, who were current or ex-cigarette smokers, and had stated the brand of their preferred e-cigarette device (*n* = 1202). Proportions using TI and independent brands were calculated and regression analysis assessed associations with sociodemographic and smoking/vaping characteristics between vapers of TI and independent products. Chi-square tests were used to analyze differences between TI and independent products.

**Results:**

Overall, 53.4% used TI products. A university education (67.6%; _adj_OR = 1.54; 95% CI, 1.140–2.088), nondaily vaping (68.2%; _adj_OR = 1.39; CI, 1.029–1.880), and cigarette dependence (moderate, strong and very strong urges to smoke) were associated with using TI vaping brands. TI products used were less likely to be refillable (“open”) than independent brands (60.9% vs. 18.3%, chi-square = 228.98, *p* < .001), more likely to use nicotine salts (16.7% vs. 8.6%, chi-square = 25.04, *p* < .001) and tobacco flavors (23.8% vs. 17.9%, chi-square = 12.65, *p* < .001).

**Conclusion:**

TI vaping products were popular in the UK, associations with product and user characteristics suggest that TI products may be less conducive to smoking cessation, although the findings were not always consistent.

**Implications:**

Consequences of regulations need to be carefully considered to ensure that independent producers are not more negatively impacted than tobacco industry producers, and to avoid reducing utility of products for smoking cessation.

## Introduction

Tobacco use is one of the world’s most serious public health threats, killing more than eight million people a year globally, and more than seven million from direct use.^1^ Cigarette smoking is the most popular form of tobacco use worldwide, and in the UK as in many other countries is most prevalent among lower-income groups.^2^

Nicotine, the primary addictive substance in tobacco, is the reason why people keep smoking, but is not the main cause of mortality and morbidity which is predominantly due to the other constituents in cigarette smoke.^3^ Therefore, products that offer a less harmful method of nicotine delivery can help to reduce smoking harms, by lowering cigarette smoke intake or enabling people to quit smoking altogether.^4^ In England, vaping products grew rapidly in popularity during the last decade, and have emerged as the most widely used quitting aid for smokers, ahead of nicotine replacement therapy (NRT), varenicline, and behavioral support.^5^ Vaping products have evolved considerably and can be separated into two categories: 1) “open” devices, where the user manually fills the device with e-liquid, and 2) “closed” devices which can sometimes resemble tobacco cigarettes, and are operable with either disposable or pre-filled cartridges or pods—for which the user has no contact with the e-liquid.^6^

There is broad agreement among researchers that e-cigarettes are less harmful than combustible tobacco cigarettes.^7^ A report by The National Academies of Sciences, Engineering, and Medicine (NASEM) found conclusive evidence that a full switch from combustible cigarettes to vaping products reduces users’ exposure to numerous toxicants and carcinogens.^8^

Concerns have been raised about the tobacco industry’s (TI) motives and intentions in the e-cigarette market. The TI has a long history of deception in the tobacco cigarette market, and has used public relations strategies since the 1950s to improve its image. Deceptive tactics employed include denying the health risks of smoking and second-hand smoke despite being aware of the dangers, manipulating nicotine levels in cigarettes, introducing additives to alter nicotine delivery, and marketing products to children.^9^ TI companies claimed “low-tar cigarettes” would help to reduce smoking harms, despite privately knowing that these products offered no health benefits and did not help smokers to quit.^10^ The TI also has a history of recruiting academics to produce research that would help resist and roll back smoking regulations, and improve the public image of smoking.^11^

The emergence of e-cigarettes, combined with the shrinking UK cigarette market,^12^ has presented TI companies with new challenges. The industry has responded by investing in the vaping market over recent years. British American Tobacco (BAT) was the first to enter the UK vaping market in 2012, with others quickly following through both the acquisition of existing e-cigarette brands, and the creation of their own e-cigarette products.^13^ Arguably, the tobacco industry could be creating vaping products that are less likely to help smokers quit (e.g. are less efficient nicotine delivery devices), and hence more likely to create dual users (people who smoke and vape^14^) or people who try their e-cigarettes and then relapse to smoking. On the other hand, the nontobacco industry manufacturers might be interested in creating vaping products that are effective smoking cessation^15^ devices but are more addictive so vapers continue to purchase them.

There is a lack of knowledge on the TI’s influence in the UK vaping market. The popularity of vaping products made by the TI, and how such products differ from those made by the independent industry, is currently unclear.

While previous research has explored overall e-cigarette use by sociodemographic groups,^16^ it has not examined the proportion of TI and independent industry products used in these groups. Nor has previous research examined differences between the products. In the light of recommendations from the WHO to restrict the types of vaping products available^17^ this will also provide evidence on whether such restrictions could favor TI or independent products. To improve understanding of the vaping market in the United Kingdom, this exploratory study addressed two research questions:

1. How popular are TI vaping products among vapers and who uses them?
2. How does vapers’ use of TI products differ from their use of independent products?

## Methods

### Participants and Design

The study used data from a single wave of a UK longitudinal online survey of smokers, ex-smokers, and nicotine users conducted in September/October 2019 by IPSOS Mori. Respondents who completed the survey were rewarded with points that could be redeemed for gift cards. Respondents were eligible to participate if they had participated in previous waves (*n* = 1720 were eligible) or for the replenishment sample if they were current smokers, ex-smokers, or e-cigarette users who had never smoked. Quotas for age, gender, ethnicity, and regions were used for the replenishment sample to enable representativeness. The final sample for the survey consisted of 1000 recontacts from previous waves and 2883 people from the replenishment sample.

To be eligible for the current study, respondents were required to be daily or nondaily e-cigarette users (*n* = 1447). Respondents were also required to confirm their smoking status. Daily and nondaily smokers were classified as dual users (smokers and vapers). Respondents who had stopped smoking completely were classified as ex-smokers. Respondents who had never smoked (*n* = 18) or did not smoke tobacco cigarettes but another kind of tobacco (e.g. pipe, cigar, or shisha) (*n* = 62) were excluded due to the small sample sizes, and for greater homogeneity of the sample being studied.

Respondents were asked which e-cigarette brand they used. Those who used a combination of e-cigarette brands (*n* = 87), those who did not know what e-cigarette brand they were using (*n* = 56), and those who provided an illegible or nonidentifiable response (*n* = 22) were excluded from the final sample.

A Total of 1202 Respondents Were Included in the Current Study

It was considered important to include a measure of smoking dependence as a predictor, as that could affect what products people were choosing to vape. As this question was not asked of ex-smokers who had quit more than 12 months ago, the sample size when including this question was reduced to 855.

King’s College London Research Ethics Committee approved this study (MRA-18/19-13459).

### Measures

#### Sociodemographic Measures

Demographic measures included gender (male/ female) and ethnicity (White/ Black, Asian, and other minority ethnic groups). Age was measured using Ipsos-defined age groups (18–24, 25–34, 35–44, 45–54, 55–65, 66 and above) and was recoded into a binary variable (18–44, 45 and above) for the regression analysis.

Socioeconomic status was measured by highest level of formal education completed, which is known to be an indicator of socioeconomic status.^18^ Six response options were provided^1^: Primary or secondary school/ vocational level 1 & 2/ trade apprenticeship,^2^ Secondary school advanced/ vocational level 3,^3^ Further education/ training college below degree level,^4^ Some university,^5^ Completed university degree,^6^ Post-graduate degree. Socioeconomic status was recoded into a binary variable (no university, some university) for the regression analysis.

Respondents were also asked where they lived, with responses collapsed into three categories: London, England excluding London, and other UK countries.

#### E-cigarette Measures

To identify whether a vaper used a TI or independent product, the following question was used: “What is the name of the brand of e-cigarettes/ vaping device that you currently use the most?” The following brands were prompted: Aspire, Blu, eGo, Eleaf, E-Lites, Gamucci, JUUL, Kangertech, Logic, Nicolites, Smok, VIP, Vivid, Vype, V2, and 88 Vape. Respondents were also given the option to type in a different brand. To establish whether the brand was owned by the TI or not, a Google search was conducted for each e-cigarette brand, followed by clicking through to the brand’s website and “About Us” page. If unsuccessful, Google searches were conducted with the e-cigarette brand name and each TI company to determine provenances. E-cigarette brands with the Independent British Vape Trade Association (IBVTA) badge^19^ on their websites were categorized as independent vape companies. Brands were also checked for TI links on the Tobacco Tactics website.^20^ When no link could be found after these search processes, the brand was categorized as independently owned. These data were coded into a binary variable, “Tobacco industry brand? Yes/ No”.

Respondents were asked which e-cigarette or vaping device they used the most and were given four response options^1^: disposable,^2^ a device using pre-filled cartridges or pods,^3^ a refillable tank device,^4^ a modular device. For some analysis, this was collapsed into “closed” (1 or 2) and “open” devices (3 or 4).

Awareness of nicotine salts was assessed through the question “Have you ever heard of e-cigarettes, cartridges, pods or e-liquids that use nicotine salts?” (response options: yes/ no/ don’t know’). Those who answered “No” or “Don’t know” were classed as “not aware”. Those who answered “Yes” were asked if they currently used nicotine salts when vaping.

In the UK, bottles of nicotine-containing liquids are restricted to 10ml.^21^ Larger bottles of nicotine-free liquid that can be filled with nicotine shots to achieve the desired strength are often described as “short fills”. Respondents were asked if they had heard of short fills and if they used them when vaping. “No” and “Don’t know” responses were classified under “not aware”. Those who answered “Yes” were asked if they currently used short fills.

Respondents were asked what nicotine strengths they used most often when vaping and were given six response options: No nicotine (0mg/ml, 0%), 1 to 8mg/ml (0.1% to 0.8%), 9 to 14mg/ml (0.9% to 1.4%), 15 to 20mg/ml (1.5% to 2.0%), 21 to 24mg/ml (2.1% to 2.4%), 25mg/ml (2.5% or more). For the regression analyses, the 21 to 24mg/ml and 25mg/ml responses were recoded into a single category, “more than 20mg”, as these responses were all greater than the legal nicotine strength limit (20mg/ml—2.0%) in the UK.

The question, “What flavors do you use in your electronic cigarettes/ vaping devices?” was asked to determine the most popular vaping flavors. The question provided 10 response options and the opportunity to write in a different flavor. “Don’t know” responses were excluded.

#### Cigarette Dependence Measure

Cigarette dependence was measured using “strength of urges to smoke”.^22^ Respondents were asked, “How much of the time have you felt the urge to smoke in the past 24 hours?”, not at all, a little of the time, some of the time, a lot of the time, almost all of the time, or don’t know. Respondents who expressed urges were asked, “In general, how strong have the urges to smoke been?”, slight, moderate, strong, very strong, and extremely strong, or don’t know. Responses were coded into a single variable—“not at all” responses from the first question were coded as 0, responses from the second question were coded 1 through 5 (1 = slight, 2 = moderate, 3 = strong, 4 = very strong, 5 = extremely strong). In both questions, “don’t know” responses were excluded.

### Analysis

#### Research Question 1

We examined what proportion of respondents used TI products. Bivariate and multivariable logistic regression was used to assess associations between the use of a TI/ independent industry vaping product and: gender, age, socioeconomic status, ethnicity, region, smoking status, vaping status, and strength of urges to smoke.

#### Research Question 2

Chi-square tests were used to assess whether there were any differences between TI and independent vaping products, by: the type of product (disposable/ pre-filled/ refillable/ modular and collapsed into open and closed devices); nicotine salts or freebase nicotine; short fills; nicotine strength; and flavors, using chi-square tests. For statistically significant and larger than 2 × 2 contingency tables, adjusted residuals >±2.58 identified cells contributing to differences between groups.^23^

All analyses were conducted using IBM SPSS Statistics Version 26.

## Results

The full sample consisted of slightly more men than women, more people in the middle age groups than the younger and older age groups, and slightly more with at least some university education. Most were from England and white. The sample consisted of more current smokers than ex-smokers, and nearly twice as many daily vapers as nondaily vapers (Table 1).

**Table 1.** Key Characteristics of the Sample With Tobacco Industry (TI) Brand and Independent Brand Breakdown (*n* = 1202)

Measure	Full sample % (*n*)	Tobacco industry brand % (*n*)	Independent brand % (*n*)
**Gender**			
Male	52.7 (634)	52.2 (335)	53.4 (299)
Female	47.3 (568)	47.8 (307)	46.6 (261)
**Age**			
18–24	9.3 (112)	11.8 (76)	6.4 (36)
25–34	22.0 (264)	24.3 (156)	19.3 (108)
35–44	23.0 (276)	23.5 (151)	22.3 (125)
45–54	22.7 (273)	20.6 (132)	25.2 (141)
55–65	14.6 (176)	12.6 (81)	17.0 (95)
66 and above	8.4 (101)	7.2 (46)	9.8 (55)
**Education**			
Prim/sec	6.2 (74)	3.9 (25)	8.8 (49)
Sec adv	20.0 (241)	17.9 (115)	22.5 (126)
FE/training/below degree level	20.8 (250)	18.2 (117)	23.8 (133)
Some university	8.5 (102)	8.3 (53)	8.8 (49)
University degree	26.5 (319)	29.8 (191)	22.9 (128)
Post-grad degree	17.5 (210)	21.7 (139)	12.7 (71)
No answer	0.5 (6)	0.3 (2)	0.7 (4)
**Ethnicity**			
White	89.4 (1075)	86.4 (555)	92.9 (520)
Black, Asian & other minority ethnic groups	9.7 (116)	12.6 (81)	6.3 (35)
No answer	0.9 (11)	0.9 (6)	0.9 (5)
**Region**			
London	16.6 (200)	21.5 (138)	11.1 (62)
England (excl. London)	68.4 (822)	65.9 (423)	71.3 (399)
Northern Ireland	1.9 (23)	1.4 (9)	2.5 (14)
Scotland	8.9 (107)	7.5 (48)	10.5 (59)
Wales	4.2 (50)	3.7 (24)	4.6 (26)
**Smoking status**			
Daily smoker	44.0 (529)	54.2 (348)	32.3 (181)
Nondaily smoker	16.4 (197)	19.2 (123)	13.2 (74)
Ex-smoker (< 12 months)	10.7 (129)	9.7 (62)	12.0 (67)
Ex-smoker (> 12 months)	28.9 (347)	17.0 (109)	42.5 (238)
**Vaping status**			
Daily vaper	65.8 (791)	56.5 (363)	76.4 (428)
Nondaily vaper	34.2 (411)	43.5 (279)	23.6 (132)
Strength of urges to smoke*			
None	7.6 (65)	5.1 (27)	11.8 (38)
Slight	8.9 (76)	6.9 (37)	12.1 (39)
Moderate	42.2 (361)	43.7 (233)	39.8 (128)
Strong	25.7 (220)	27.8 (148)	22.4 (72)
Very strong	10.3 (88)	11.8 (63)	7.8 (25)
Extremely strong	5.3 (45)	4.7 (25)	6.2 (20)

Each column provides column percentages.

* Ex-smokers who quit more than 12 months ago (n = 347) were not asked this question.

### How Popular are TI Vaping Products Among Vapers and Who Uses Them?

Of the 1202 vapers included in the study, 53.4% (*n* = 642) were using a TI product, while 46.6% (*n* = 560) were using an independent product. Table 1 describes demographic, vaping, and smoking characteristics for those using a TI or independent brand.

There were 61 brands reported in total, 12 from the TI and 49 from the independent industry.

The 10 most popular brands were: Blu (232), Aspire (138), Vype (114), Smok (85), E-Lites (73), JUUL (57), 88Vape (54), Eleaf (53), Logic (53) and Innokin (43). Five of these brands (Blu, Vype, E-Lites, JUUL, and Logic) are owned by tobacco industry companies. Blu is owned by Imperial Brands, Vype by British American Tobacco, JUUL by Altria Group, and both E-Lites and Logic by Japan Tobacco International (Supplementary Table A1).

Regression analyses indicated that university-educated vapers were more likely to use TI vaping products, vapers in England and “other GB countries” were less likely to use TI vaping products than vapers in London. Ex-smokers were less likely to use TI vaping products than current smokers. Nondaily vapers were more likely to use TI vaping products than daily vapers. In bivariate analyses only, older vapers were less likely to use TI vaping products and vapers from Black, Asian, and other minority ethnic groups more likely to use TI products. Gender was not associated with using a TI vaping brand (Table 2).

**Table 2.** Associations Between Sociodemographics, Smoking/Vaping Status and Use of Tobacco Industry Brands (*n* = 1185*)

			Unadjusted (bivariate)			Adjusted (multivariable)		
Variable	n	% using TI vape	OR	95% CI	*p* value	OR	95% CI	*p* value
**Gender**								
Male (ref)	623	53.0	1	1	Ref	1	1	Ref
Female	562	54.1	1.05	0.832–1.315	.699	1.09	0.849–1.399	.501
**Age**								
18–44 (ref)	638	58.9	1	1	Ref	1	1	Ref
45 and above	547	47.2	**0.62**	**0.494–0.783**	**<.001**	0.94	0.723–1.220	.637
**Education**								
No university (ref)	562	45.4	1	1	Ref	1	1	Ref
Some university	623	60.8	**1.87**	**1.484–2.356**	**<.001**	**1.59**	**1.236–2.043**	**<.001**
**Ethnicity**								
White (ref)	1069	51.7	1	1	Ref	1	1	Ref
Black, Asian and other minority ethnic groups	116	69.8	**2.16**	**1.427–3.268**	**<.001**	1.38	0.886–2.148	.155
**Region**								
London (ref)	195	68.7	1	1	Ref	1	1	Ref
Engl exc London	814	51.6	**0.49**	**0.348–0.677**	**<.001**	**0.64**	**0.447–0.909**	**.013**
Other GB Countries	176	45.5	**0.38**	**0.248–0.580**	**<.001**	**0.49**	**0.315–0.774**	**.002**
**Smoking status**								
Current smoker (ref)	716	65.1	1	1	Ref	1	1	Ref
Ex-smoker	469	35.8	**0.30**	**0.235–0.382**	**<.001**	**0.38**	**0.294–0.499**	**<.001**
**Vaping status**								
Daily vaper (ref)	781	46.1	1	1	Ref	1	1	Ref
Nondaily vaper	404	67.8	**2.47**	**1.916–3.170**	**<.001**	**1.77**	**1.353–2.324**	**<.001**

Other GB countries = Northern Ireland, Scotland and Wales. CI: confidence interval; Ref: reference group; OR: odds ratio. Significant associations (*p* < .05) are highlighted in bold.* *n* for Table 2 is reduced by 17 due to missing data (ethnicity: *n* = 11, education *n* = 6)

The secondary analysis including urges to smoke and thereby excluding long-term ex-smokers found broadly similar associations as the primary analysis for gender, education, ethnicity, region, and vaping frequency. (Table 3) The associations between smoking status and age and use of TI brands were attenuated. Urges to smoke were also associated with using a TI brand with those reporting stronger urges to smoke more likely to be using a TI brand.

**Table 3.** Associations Between Sociodemographics, Smoking/Vaping Status and Urges to Smoke With Use of Tobacco Industry Brands Among Smokers and Ex-smokers Who Stopped Within the Last 12 Months, Long-term Ex-smokers Excluded (*n* = 843.).

			Unadjusted (bivariate)			Adjusted (multivariable)		
Variable	n	% using TI vape	OR	95% CI	*p* value	OR	95% CI	*p* value
**Gender**								
Male (ref)	448	62.7	1	1	Ref	1	1	Ref
Female	395	62.0	0.97	0.734–1.283	.835	1.06	0.785–1.422	.717
**Age**								
18–44 (ref)	533	63.4	1	1	Ref	1	1	Ref
45 and above	310	60.6	0.89	0.667–1.186	.424	1.09	0.796–1.494	.589
**Education**								
No university (ref)	362	55.5	1	1	Ref	1	1	Ref
Some university	481	67.6	**1.67**	**1.259–2.212**	**<.001**	**1.54**	**1.140–2.088**	**.005**
**Ethnicity**								
White (ref)	738	60.8	1	1	Ref	1	1	Ref
Black, Asian and other minority ethnic groups	105	73.3	**1.77**	**1.121–2.796**	**.014**	1.52	0.938–2.462	.089
**Region**								
London (ref)	161	73.9	1	1	Ref	1	1	Ref
Engl exc London	556	61.2	**0.56**	**0.376–0.821**	**.003**	0.67	0.443–1.003	.052
Other GB Countries	126	53.2	**0.40**	**0.244–0.658**	**<.001**	**0.48**	**0.285–0.800**	**.005**
**Smoking status**								
Current smoker (ref)	716	65.1	1	1	Ref	1	1	Ref
Ex-smoker	127	47.2	**0.48**	**0.328–0.703**	**<.001**	0.71	0.459–1.111	.135
**Vaping status**								
Daily vaper (ref)	488	58.2	1	1	Ref	1	1	Ref
Nondaily vaper	355	68.2	**1.54**	**1.155–2.049**	**.003**	**1.39**	**1.029–1.880**	**.032**
**Strength of urges to smoke**								
Not at all (ref)	65	41.5	1	1	Ref	1	1	Ref
Slight	76	48.7	1.34	0.685–2.603	.396	1.06	0.527–2.120	.877
Moderate	356	64.6	**2.57**	**1.499–4.404**	**.001**	**1.84**	**1.015–3.337**	**.044**
Strong	215	67.4	**2.92**	**1.649–5.154**	**<.001**	**2.19**	**1.166–4.096**	**.015**
Very strong	86	72.1	**3.64**	**1.838–7.192**	**<.001**	**2.64**	**1.249–5.580**	**.011**
Extremely strong	45	55.6	1.76	0.817–3.790	.149	1.24	0.545–2.810	.611

Other GB countries = Northern Ireland, Scotland and Wales. CI: confidence interval; Ref = reference group; OR: odds ratio. Significant associations (*p* < .05) are highlighted in bold.

### How Do TI Products Differ From Independent Products?

TI products appeared more likely to be disposables or cartridges/pods devices, while independent devices were more likely to be refillable or modular devices (Table 4, all groups contributed to the difference). Overall, TI products were more likely to be “closed” devices (60.9% versus 18.3% of independent products, χ² ^1^ = 228.98, *p* < .001).

**Table 4.** Characteristics of Tobacco Industry and Independent Vaping Products (*n* = 1202)

Variable	Total % (n)	Tobacco industry brand % (*n*)	Independent industry brand % (*n*)	χ² (d.f.), *p*
**Device type** ^a^				χ² (3) = 245.35, *p* < .001
Disposable	8.5 (102)	**11.2 (72)**	**5.4 (30)**	
Cartridges/Pods	32.5 (391)	**49.7 (319)**	**12.9 (72)**	
Refillable tank system	43.8 (527)	**31.8 (204)**	**57.7 (323)**	
Modular	14.3 (172)	**6.2 (40)**	**23.6 (132)**	
Don’t know	1.1 (10)	7	3	
**Nicotine salt status**				χ² (2) = 25.04, *p* < .001
Using	12.9 (155)	**16.7 (107)**	**8.6 (48)**	
Not using	14.1 (169)	**10.9 (70)**	**17.7 (99)**	
Not aware / don’t know	73.0 (878)	72.4 (465)	73.8 (413)	
**Short fill status**				χ² (2) = 4.41, *p* = .110
Using	15.4 (185)	14.2 (91)	16.8 (94)	
Not using	17.8 (214)	16.4 (105)	19.5 (109)	
Not aware / don’t know	66.8 (803)	69.5 (446)	63.7 (357)	
**Nicotine strength** ^b^ ^,^ ^c^				χ² (5) = 21.87, *p* = .001
No nicotine	7.2 (87)	6.4 (39)	8.1 (48)	
1 to 8 mg/ml (0.1% to 0.8%)	35.7 (429)	**31.8 (195)**	**39.7 (234)**	
9 to 14 mg/ml (0.9% to 1.4%)	26.5 (318)	**30.5 (187)**	**22.2 (131)**	
15 to 20 mg/ml (1.5% to 2.0%)	24.6 (296)	23.5 (144)	25.8 (152)	
21 to 24 mg/ml (2.1% to 2.4%)	2.7 (33)	3.6 (22)	1.9 (11)	
25 mg/ml (2.5%) or more	2.7 (32)	3.6 (22)	1.7 (10)	
Don’t know	0.6 (7)	0.7 (4)	0.5 (3)	
**Flavor** ^d^				
Fruit	24.3 (482)	22.6 (240)	26.3 (242)	χ² (1) = 4.23, *p* = .040
Tobacco	21.0 (417)	23.8 (252)	17.9 (165)	χ² (1) = 12.65, *p* < .001
Menthol/Mint	18.1 (358)	17.0 (180)	19.3 (178)	χ² (1) = 2.01, *p* = .156
Chocolate, dessert and sweet candy	9.1 (180)	7.5 (79)	11.0 (101)	χ² (1) = 7.71, *p* = .005
Vanilla	8.2 (162)	8.5 (90)	7.8 (72)	χ² (1) = 0.346, *p* = .556
Tobacco and menthol	7.0 (139)	8.2 (87)	5.6 (52)	χ² (1) = 5.32, *p* = .021
Coffee	6.2 (122)	6.8 (72)	5.4 (50)	χ² (1) = 1.72, *p* = .190
Drink or no flavor	6.1 (121)	5.7 (60)	6.6 (61)	n/a

Each column provides column percentages. Bold font indicates cells associated with adjusted residuals greater than ±2.58 (α = 0.01)

^a^ Chi-square excludes *n* = 10 who responded don’t know

^b^ Missing data for 64 respondents

^c^ Chi-square excludes *n* = 7 who responded don’t know

^d^ *N* is greater for flavors as respondents could choose more than one flavor

Vapers using TI products were more likely to be using nicotine salts, however, most vapers (73.0%) were either not aware of nicotine salts or did not know if they were using them (Table 4). Among vapers who were aware, the difference remained significant (χ² ^2^ = 24.66, *p* < .001).

Most vapers (66.8%) were not aware of short fills and similar proportions of those using TI and independent products used short fills (Table 4). Results were similar among vapers who were aware (χ² ^2^ = 26.28, *p* < .001).

Products with a lower nicotine strength appear to be more likely to be independent brands. More than 5% of vapers reported using nicotine strengths over the UK legal limit. Vapers who did not know what nicotine strength they were vaping were more likely to be using TI brands.

Significant associations between flavor type and brand provenance were found for four flavors. “Chocolate, dessert, and sweet candy” and “Fruit” flavored products were more likely to be from independent brands, whereas “Tobacco” and “Tobacco and Menthol” flavored products were more likely to be from TI brands (Table 4).

## Discussion

Just over half of vapers in this survey in the United Kingdom were using TI products. Those who had some university education, were living in London, vaped less than daily, concurrently smoked, and had moderate to strong urges to smoke were more likely to be using a TI brand when controlling for other sociodemographic and smoking behaviors. TI products that were used were generally more likely to be closed systems (with cartridges/pods much more popular than disposables), contain nicotine salts (where users were aware of them), and appeared less likely to have a lower nicotine level (1 to 8mg/ml); they were also more likely to be tobacco or tobacco and menthol-flavored than independent products.

The findings indicate that the TI has established a firm presence in the UK e-cigarette market since its entry in the early 2010s. Therefore, concerns about the TI’s ambitions and motives in it are valid and merit research.

It is important regulations are informed by the effectiveness of different types of device and their health impacts, rather than the type of manufacturer. Research is emerging on the relative effectiveness of different devices, such as closed vs open systems, but more research is needed in this area and on other characteristics of devices such as flavors and how nicotine is delivered. Concerns have previously been raised that the TI wants people who vape to vape and smoke (dual users), as opposed to just vape^14^ and that the TI was investing in least effective devices.^24^ In our study, the TI products used were less likely to be open systems, more likely to have some tobacco flavoring, and more likely to be used by nondaily vapers, which may suggest that TI products may reduce the likelihood of smoking cessation, based on some but not all evidence.^25–27^ In line with this, the study showed that TI brands were associated with stronger urges to smoke, which suggests they may be less likely to help with nicotine withdrawal, as well as concurrent smoking. However, users of TI products may also be more likely to experience stronger urges to smoke if, for example, they are using the products for temporary abstinence only. It has also been shown that nondaily vaping is associated with reduced likelihood of quitting smoking^25,28^ and there is recent evidence that nontobacco flavors are associated with increased likelihood of transitioning away from cigarettes.^29^

TI products used in this study were more likely to contain nicotine salts, and less likely to have a lower nicotine strength, which may be associated with greater smoking cessation effectiveness but also increased addictiveness. More research is needed to assess the impact of these different characteristics on quitting smoking.

Finally, certain sociodemographics were associated with using a TI product. It is unclear why those living in London would be more likely to use TI products, although evidence elsewhere suggests independents are more dominant in the north of England (Frances Thirlway, University of York, personal communication). A university education predicted using a TI brand, which could be due to pricing structure. Those on lower incomes, where smoking is most prevalent, may be less able to afford TI products if they are priced higher. It is possible that TI marketing strategies underpin their greater usage by younger vapers and vapers from Black, Asian and other minority ethnic groups but as these relationships were only identified in bivariate analyses in our study, they should be explored in further research.

### Strengths and Limitations

This study has, to our knowledge, provided the first insights into the popularity of TI products among vapers in the UK, the characteristics of TI products used, and the characteristics of those who are using them.

An important strength of the study was that it came from a general population survey and looked at all vapers, as opposed to looking at vaping products sales data, which do not include online or vape shop purchases.

The manufacturer of the products was not always easy to identify, although it was ultimately possible to classify 98% of the products named.

This study was limited in that some analyses did not include ex-smokers who had quit more than 12 months ago, because the survey did not ask them about dependence. These ex-smokers accounted for more than a quarter of the full sample, and more than two-thirds of all ex-smokers. We did not collect duration of use of specific devices in this study, so it’s possible that over time vapers gravitate towards non-TI products as suggested in our study for those who had been ex-smokers for one year or less compared with ex-smokers who used them for more than one year.

As smokers and recent ex-smokers are a majority of our sample, the popularity of TI products could be explained if TI e-cigarettes are more likely to be available, or promoted, at the point of sale where smokers typically purchase their tobacco cigarettes. Additionally, we did not include vapers who were never smokers who represent a very small proportion of the vaper population in the UK.^5^

The study also excluded respondents who used a combination of brands (6% of eligible respondents). For example, a vaper could have an independent refillable tank, and be filling it with e-liquid manufactured by a TI brand or be mixing TI and independent e-liquids together. Future research could explore what e-liquid brands people are using in tank systems.

Furthermore, it is not certain that this survey was representative of all vapers in the UK although the recruitment used quotas to obtain a good representation. This study’s findings may not be applicable to vaping markets in other countries.

### Implications for Research and Practice

Some of the responses indicated that some vapers were unfamiliar with vaping terminology used by researchers, as shown in the question asking about e-cigarette device type. This highlights the need to constantly pilot survey questions.^30^

Requiring the manufacturer to be marked clearly on the product packaging would make distinction of TI and independent products clearer and allow smokers/vapers a choice of whether they wish to use TI products.

Future research could explore price differences between TI and independent brands, and the associations between TI brands, cigarette dependence, and nondaily vaping.

Further research is needed to explain why pre-filled cartridges/pods are more popular with TI vapers, and whether the industry has any ulterior motives with these products. This is particularly important, given that the popularity of these types of vaping products increased for the first time in 2019, suggesting a greater influence of the TI on the UK vaping market.^31^ More studies are needed into why increased cigarette dependence and nondaily vaping predicted using a TI brand.

This exploratory study suggests current claims from TI companies that they want to be involved in reducing smoking harms, and help to create a smoke-free future^32^ require further research on the nature of the products, how they are used in the real world and the extent to which they help smokers to stop. Studies exploring the impact of other characteristics associated with TI products—salts, tobacco, or tobacco and menthol flavoring—could also improve our understanding of the TI’s ambitions and motives in the UK e-cigarette market.

Finally, the present study indicates that restricting vaping devices to closed systems as recently recommended by the WHO^17^ could have a number of unintended adverse consequences. It would likely favor the tobacco industry over independent producers of vaping products. TI brands were associated with nondaily vaping, tobacco flavors, and nicotine salts. Nondaily vaping and tobacco flavors are characteristics that make complete switching (smoking cessation) less likely and perpetuate dual use. Banning nontobacco flavors may benefit the tobacco industry. We also recognize that e-cigarette characteristics that increase quitting may also increase their uptake by never smokers, but this is beyond the scope of this paper.

## Conclusions

Just over half of vapers in this survey from the UK were using products from the TI, indicating that TI products are popular among vapers. These TI products were predominantly closed systems (disposables and cartridges/pods). Restricting vaping products to closed systems would favor TI products. When controlling for sociodemographic and smoking measures, greater urges to some cigarettes and nondaily vaping independently predicted using a TI brand which suggests TI brands may be less helpful with smoking cessation, but more research is needed here. Having some university education predicted using a TI brand, which may reflect the pricing structure but also suggests that such products would not be used by lower-income groups where smoking is most prevalent.

## Supplementary Material

A Contributorship Form detailing each author’s specific involvement with this content, as well as any supplementary data, are available online at https://academic.oup.com/ntr.

ntab253_suppl_Supplementary_Table

ntab253_suppl_Supplementary_Taxonomy_Form

## Data Availability

Data availability Statement: Data are available from the corresponding author upon request.
