# Supplementary material for: The Use of Tobacco Industry Vaping Products in the UK and Product Characteristics: A Cross-Sectional Survey
Source: Nicotine Tob Res. 2021 Dec 9;24(7):1003–11. doi: 10.1093/ntr/ntab253 (PMC9199949; doi:10.1093/ntr/ntab253)
Supplement: ntab253_suppl_Supplementary_Table [file ntab253_suppl_supplementary_table.docx]

Supplementary Table

| Table A1 | | |
| --- | --- | --- |
| *Breakdown of e-cigarette brands used by vapers (n = 1202)* | | |
| Ownership | Brand | n |
| **Tobacco industry** | **Altria** |  |
|  | JUUL | 57 |
|  | PAX | 1 |
|  | V2* | 10 |
|  | Vapour2* | 1 |
|  | **British American Tobacco** |  |
|  | 10Motives | 24 |
|  | VIP | 38 |
|  | Vype | 114 |
|  | **Imperial Brands** |  |
|  | Blu** | 232 |
|  | **Japan Tobacco International** |  |
|  | E-Lites (now owned by Logic) | 71 |
|  | Logic | 53 |
|  | **Philip Morris International** |  |
|  | Nicolites (now Nicocigs) | 20 |
|  | Vivid*** | 21 |
|  |  |  |
| **Independent industry** | 88Vape | 54 |
|  | Aquavape | 1 |
|  | Aspire | 138 |
|  | Council of Vapers | 1 |
|  | Dot | 1 |
|  | Dovpo | 1 |
|  | eGo | 40 |
|  | Ehpro | 1 |
|  | Eleaf | 53 |
|  | FreeMax | 2 |
|  | Gamucci | 14 |
|  | Geekvape | 3 |
|  | Gower | 1 |
|  | GS (Greensound) | 1 |
|  | Hoxton | 1 |
|  | Imecig | 1 |
|  | Innokin | 43 |
|  | Jac Vapour | 5 |
|  | Jenson | 1 |
|  | Jomo (JomoTech) | 1 |
|  | JoyeTech | 4 |
|  | juNo | 1 |
|  | Justfog | 1 |
|  | Kamri | 1 |
|  | Kangertech | 40 |
|  | Kik | 2 |
|  | Magic Mist | 2 |
|  | Matchless | 1 |
|  | Miniciggy | 1 |
|  | Mirage | 1 |
|  | MV (MultiVape) | 1 |
|  | OBS | 1 |
|  | OK | 3 |
|  | Orchid | 1 |
|  | Smok | 85 |
|  | Socialites (now EDGE Vaping) | 1 |
|  | SteamCrave | 1 |
|  | Suicide Mods | 1 |
|  | TECC | 7 |
|  | Totally Wicked | 17 |
|  | Vampire Vape | 1 |
|  | VapeHQ | 1 |
|  | VapeMate | 1 |
|  | Vaporesso | 12 |
|  | Vapouriz | 4 |
|  | Vapourlites | 2 |
|  | Vapoursson | 2 |
|  | Vision Spinner | 1 |
|  | Voopoo | 1 |
| * V2 and Vapour2 are both now owned by JUUL | | |
| ** Includes 1 Skycig user – Skycig has rebranded as Blu | | |
| *** Vivid is a sub-company of Nicocigs | | |
